# Recovery of the hibernating cavernosum by penile revascularization

**DOI:** 10.1080/20905998.2024.2333675

**Published:** 2024-03-23

**Authors:** Yasuo Kawanishi, Takeshi Miyake, Masahito Yamanaka

**Affiliations:** Department of Urology, Takamatsu Red Cross Hospital, Takamatsu, Kagawa, Japan

**Keywords:** Cavernous function recovery, erectile dysfunction, trabecular nourishment, vascular surgery, hibernation, hibernating cavernosum

## Abstract

**Objective:**

Chronic ischemia-related cavernous dysfunction is considered irreversible. However, in certain patients, cavernous function appears to recover with penile revascularization. In this study, we investigated a potential cavernous dysfunction reversibility from a clinical perspective.

**Patients and Methods:**

We involved 93 young patients in the study with arterial erectile dysfunction (ED) (median age: 30 years). Erectile function tests were performed according to the standard operating procedures of the International Society of Sexual Medicine. Among the participants, 63 and 30 displayed pure arteriogenic and mixed vasculogenic (due to both arterial insufficiency and cavernous dysfunction) ED, respectively. Penile revascularization was performed by anastomosing the inferior epigastric artery to the dorsal artery. The ED treatment success was considered from a score of at least 24 on the International Index of Erectile Function-6.

**Results:**

Our results proved that penile revascularization cured 92.1% and 73.8% of the patients with pure arteriogenic and mixed vasculogenic ED, respectively (Kaplan-Meier method, log-rank test: no significant difference). The required time for curing 50% of the patients was 10.5 and 10.0 months for pure arteriogenic and mixed vasculogenic ED, respectively, indicating no recovery delay in patients with mixed vascular ED. Furthermore, the cavernous dysfunction degree did not influence cavernous function recovery.

**Conclusion:**

Penile revascularization cured ED in 73.8% of the patients with mixed vasculogenic ED. Cavernous dysfunction appears to be reversible in certain cases. Furthermore, we observed no delay in functional recovery compared to participants with healthy cavernous function. These two discoveries suggest that cavernous function recovery after penile revascularization is similar to the concept of hibernating myocardium in ischemic myocardium. Although cavernous dysfunction is considered irreversible, it could be reversed in multiple cases with blood flow restoration to the cavernous tissue.

## Introduction

Insufficient arterial flow to the penis could cause ischemic or arteriogenic erectile dysfunction (ED). In addition, penile ischemic conditions could result in cavernous dysfunction [1]. Penile revascularization surgery could prove efficient in arteriogenic ED treatment among young patients with no cavernous dysfunction. However, surgery is not recommended in the case of cavernous dysfunction [2] as cavernous dysfunction treatments are not yet complete [3–5].

Nevertheless, in the myocardium, on which several ischemic condition-related studies focus, an interesting concept, i.e. the ‘hibernating myocardium’ was proposed by Rahimtoola in 1985 [6]. The essence of the concept is that under certain ischemic conditions, myocardial function declines, but the myocardium survives without necrosis and could resume its function once blood flow is restored. Ischemic degree and duration in the corpus cavernosum are patient-dependent. Fibrosis and necrosis do not occur in all cases. A reversible condition (hibernating cavernosum) might thus exist, in which the cavernous function recovers without delay once the blood supply to the cavernosal tissues resumes.

This study aimed at assessing corpus cavernosum function reversibility using erectile function recovery as an indicator. With the approval of the ethics committee, we revascularized the dorsal artery of the penis in a patient with cavernous dysfunction-complicated arteriogenic ED. Our intervention yielded cavernous function restoration.

## Methods

We conducted this cohort study in a single institution, running erectile function tests according to the standard operating procedures of the International Society for Sexual Medicine [7]. We performed a subjective assessment using the International Index of Erectile Function (IIEF) questionnaire, a blood test, and a duplex Doppler ultrasound test of the cavernous arteries using alprostadil alfadex.

**Figure 1.**
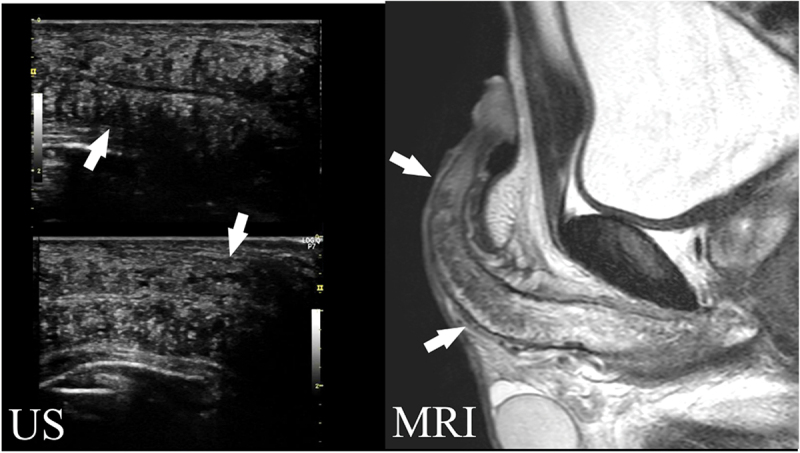
A case with cavernous body fibrosis (>20 mm).

To evaluate the cavernous function were performed dynamic infusion cavernosometry and cavernosography (DICC) with alprostadil redoing schedule in all cases. We inserted two butterfly needles into the corpus cavernosum: one to measure intracavernous pressure and the other for injection. We injected saline using a servo-controlled pump and measured the required perfusion rate for the maintenance of a 90-mmHg intracavernous pressure. We limited the maximum flow rate to 120 mL/min to avoid circulatory system overload. Prior to the puncture, we used local anesthetic tape on patients who suffered from severe pain during previous intracavernous injections to prevent pain-related sympathetic inhibition. We defined optimal cavernous function at a maintenance flow rate of 20 mL/min and cavernous pressure of 90 mmHg. We conducted selective internal pudendal angiography for a definitive diagnosis in all patients included in the study based on the following criteria: suboptimal response to 10-μg intracavernous alprostadil alfadex injection, angiographically confirmed local disease of the internal pudendal artery or its distal branches, and penile revascularization of the dorsal artery. The exclusion criteria were as follows: being diagnosed with diabetes, neurological disease, depression, schizophrenia, Peyronies disease, and endocrine abnormalities; cases in dispute due to industrial or traffic accidents; cases with cavernous fibrosis with a long diameter of 20 mm on MRI (Figure 1).

Ultrasonography (US) highlights the rough hyperechoic lesion within the cavernous body (white arrows).

Magnetic resonance imaging (MRI): T2WI low signal intensity lesion could be observed with irregular margins in the corpus cavernosum on both sides from the tip to the base of the corpus cavernosum (white arrows). According to the exclusion criteria, this case was excluded from the indication for penile revascularization.

## Surgical techniques

**Figure 2.**
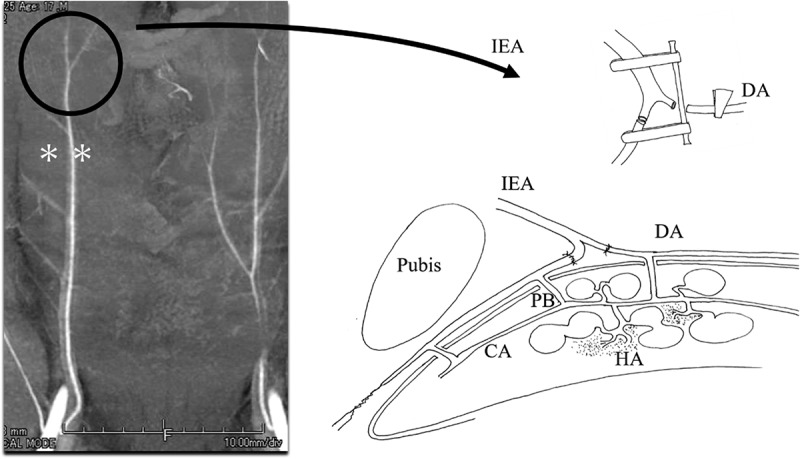
Microvascular anastomosis between the distal end of the inferior epigastric artery and the dorsal artery of the penis.

For penile revascularization surgery, we selected a surgical method that could restore cavernous tissue nourishment [8]. Specifically, we anastomosed the inferior epigastric artery to the dorsal artery of the penis to increase blood flow in the cavernous and helicine arteries. Unlike the anastomosis of the inferior epigastric artery to the deep dorsal vein, this revascularization method increases both pressure in the cavernous sinus and blood flow in the circulatory system that feeds the trabeculae [9,10]. Under magnifying surgical glasses, we exposed and dissected the inferior epigastric artery. The dissection was continued cephalad for 12–13 cm to free two major branches (Figure 2). We clipped and divided them, then lowered them down to the internal inguinal ring and passed them through the inguinal canal to the base of the penis. Next, we performed the operation under a microscope. We detached the penile dorsal nerve and accompanying veins from the dorsal artery of the penis. During this operation, we restored the blood flow in the corpus cavernosum via the branches of the dorsal artery of the penis, these branches should thus be preserved as much as possible. We cut the dorsal artery of the penis and anastomosed it end-to-end with the harvested inferior epigastric artery end using an 11–0 polyamide suture (single-armed, 80 μm, using a 3-mm needle). We performed vascular anastomosis both in the proximal and distal directions of the dorsal artery of the penis [11]. After bidirectional anastomosis, we performed indocyanine green video angiography to confirm anastomosis patency.

The inferior epigastric artery originates from the external iliac artery, runs cranially, and gives off several branches to the rectus abdominis muscle. The inferior epigastric artery often bifurcates near the umbilicus (**). To perform vascular anastomosis in both the proximal and distal directions of the dorsal artery of the penis, we used the bifurcation of the distal end of the hypogastric artery for vascular anastomosis. After vascular anastomosis, the blood flow within the cavernous artery increases through the penetrating arteries. The helicine arteries branch off from the cavernous artery and open into the cavernous sinus. Apart from these arteries, several helicine arteries terminate in capillaries and feed the trabeculae (cavernous tissue).

IEA: inferior epigastric artery

DA: dorsal artery of the penis

PB: penetrating branch

CA: cavernous artery

HA: helicine artery.

We administered alprostadil alfadex (5 μg/h) intravenously and Aspirin (100 mg/day) for 3 consecutive days and at least 1 year after surgery, respectively.

Our primary endpoint was cavernous function recovery over time after the surgery. We considered postoperative erectile function recovery a proof of cavernous function recovery as cavernous dysfunction is a significant cause of ED and erectile function is unlikely to recover with maintained cavernous dysfunction. We used IIEF-6 [12], an abridged IIEF version [13], to evaluate surgical outcomes. IIEF is a multidimensional self-report instrument consisting of 15 items and five domains of sexual function. We used a 24-point IIEF-6 score cutoff. Scoring 24 IIEF-6 points roughly represents ‘optimal’ erection during intercourse most of the time and ‘high’ confidence in maintaining an erection. Our secondary endpoint was to determine cavernosal dysfunction severity, i.e. how flow maintenance at 90 mmHg in DICC affects postoperative recovery.

## Statistical analysis

We evaluated our surgical results using the Kaplan – Meier method. Patients who started other ED treatments or were not cured at the time of the survey were considered censored cases as of the start date of that treatment or the date of their last visit, respectively. We performed the log-rank test and logistic regression analysis using IBM SPSS Statistics version 28.0.1.0. (USA) and set the significance level at *p* < 0.05.

## Results

### Patient characteristics

Between 2005–2023, 93 patients met our inclusion criteria. Table 1 summarizes the characteristics of these 93 patients. We observed no statistical differences between the two groups, except for the preoperative DICC parameter. Of these 93 patients, 63 and 30 exhibited pure and cavernous dysfunction-related arteriogenic ED, respectively. We only revascularized the dorsal artery of the penis in both groups without attempting therapeutic venous leak treatment.

**Table 1.** Cavernous function-related patient background.

		Optimal cavernous function	Cavernous dysfunction
Number of case		63 cases	30 cases
Median age at surgery (range), years		30.5 [14–]47)	31.0 [14–]45)
Diabetes mellitus		0	0
Current smoking habit		0	0
Hypertension		0	0
Preoperative IIEF-6, (range), points		9.1 ± 6.3 [1–]23)	6.5 ± 4.5 [1–13–18]
Response to intracavernous 10-µg injection of prostaglandin E1 (erection hardness score)		2.5 ± 0.8 [1–3]	2.0 ± 0.7 [1–3]
Mean peak systolic velocity of cavernous arteries, (range), cm/s		23.4 ± 7.7 (6.7–37.7)	21.9 ± 9.0 (0–42.5)
Mean end-diastolic velocity of cavernous arteries, (range), cm/s		3.4 ± 3.4 (−2.1–11.6)	4.2 ± 5.0 (−0.7–22.7)
Flow rate to maintain an intracavernous pressure of 90 mmHg at dynamic infusion cavernosometry, mL/min	<20 20 ≤ < 30 30 ≤ < 100 100 ≤	63 cases 0 cases 0 cases 0 cases	0 cases 13 cases 9 cases 8 cases

## Primary endpoint

**Figure 3.**
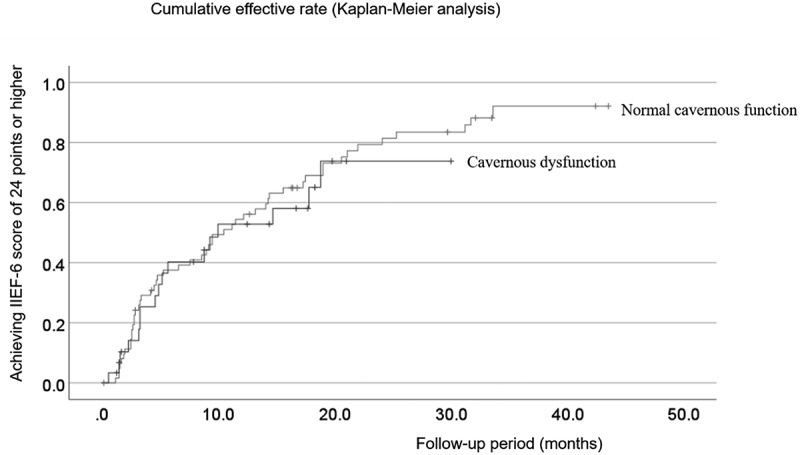
Cumulative rate of microvascular penile revascularization efficacy.

For those who were cured, the phase until recovery was defined as the follow-up period, ranging between 0.6–33.6 (median: 5.8) months. For patients who were not cured at the time of the study, the follow-up ranged between 1.2–43.5 (median: 16.4) months. The Kaplan – Meier method indicated that the final cumulative efficacies of the patient groups with optimal cavernous function and cavernous dysfunction were 92.1% and 73.8%, respectively (Figure 3). We observed no statistical difference based on the log-rank test. Reaching cumulative effectiveness of 50% took 10.5 ± 1.7 and 10.0 ± 4.2 months in the patient groups with optimal cavernous function and cavernous dysfunction, respectively. In patients with optimal cavernous function, the pre- and postoperative mean values of the erection hardness score [15] after prostaglandin E1 intracavernous injections were 2.5 ± 0.8 (1–3, *n* = 63) and 3.9 ± 0.3 (3–4, *n* = 14). In patients with cavernous dysfunction, the pre- and postoperative mean values were 2.0 ± 0.7 (1–3, *n* = 30) and 3.7 ± 0.3 (3–4, *n* = 9).

The Y- and X-axes refer to the percentage of patients who achieved an IIEF6 score of ≥ 24, calculated using the Kaplan – Meier method and the postoperative follow-up period in months, respectively. Our log-rank test did not detect any statistically significant difference between the two patient groups.

## Secondary endpoint

**Figure 4.**
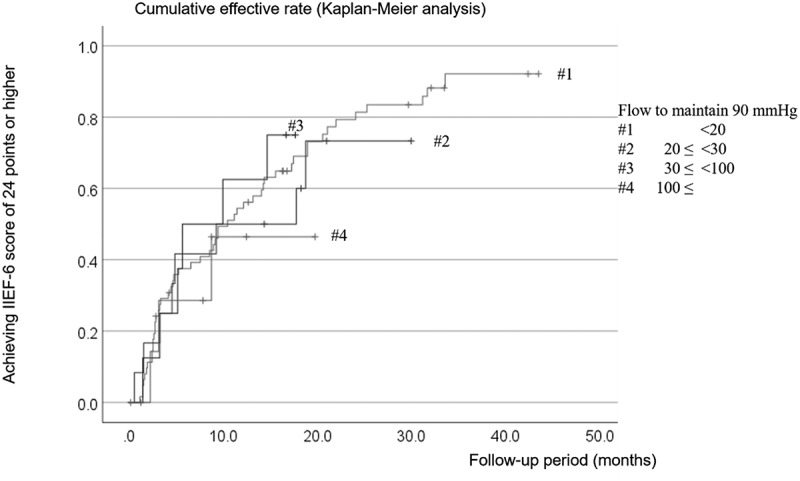
Degree of cavernosal dysfunction (flow to maintain erection in DICC) and penile microvascular revascularization cumulative efficacy rate.

To clarify how the cavernosal dysfunction degree impacted recovery, we divided the patients into four groups according to the maintenance flow rate at an intracavernous pressure of 90 mmHg. Group #1 comprised patients with a maintenance flow rate below 20 mL/min, i.e. those with optimal cavernous function. We divided patients with cavernosal dysfunction with a maintenance flow rate of 20 mL/min into three groups (Figure 4). Figure 4 represents the cumulative efficacy rate for each group calculated using the Kaplan – Meier method. We observed no statistical difference in the cumulative efficacy rate determined by the log-rank test. The 12-month cumulative efficacy rates for groups 1, 2, 3, and 4 were 54.4 ± 6.4%, 50.0 ± 14.4%, 62.5 ± 17.1%, and 46.4 ± 20.1%, respectively.

The Y- and X-axes refer to the percentage of patients who achieved an IIEF6 score of ≥ 24, calculated using the Kaplan – Meier method and the postoperative follow-up period in months, respectively. Our log-rank test did not detect any statistically significant differences between the patient groups.

## Complications

We classified the adverse events according to the Common Terminology Criteria for Adverse Events (CTCAE) v5.0 [16], most being transient and grade-1 including penile edema, discomfort, and perineal pain (Table 2). We registered no cases of penile shortening. One patient developed retinal hemorrhage, which was considered a grade-2 adverse event. Another patient had an anastomotic rupture 5 weeks postoperatively due to a trauma requiring emergency surgery, which was considered a grade-3 adverse event.

**Table 2.** Adverse events.

CTCAE v5.0 code	CTCAE v5.0 Term	Grade	Clinical course
10018146	Genital edema	Grade 1	Disappeared within 3 days.
10034310	Penile pain	Grade 1	Disappeared within 1 month.
10055322	Postoperative hemorrhage	Grade 3	Accidental rupture of the anastomosis 5 weeks after surgery due to a collision with a boy. Reparative surgery was performed at our hospital within 24 h.
10055322	Postoperative hemorrhage	Grade 1	Resolved within 3 days.
10061339	Perineal pain	Grade 2	Continued for >3 months, no medication needed after the second month.
10038604	Reproductive system – Other	Grade 2	Discomfort of glans at erection, resolved within 3 months.

CTCAE: Common Terminology Criteria for Adverse Events.

## Discussion

Cavernous dysfunction is a serious cause of ED with the clinical manifestation of corporal veno-occlusive dysfunction. The possibility of functionally recovering a damaged corpus cavernosum was clearly described by El-Sakka AI already in 2011 [3]. However, since then, research advances on cavernous recovery have been limited. In this study, we investigated cavernous function recovery after penile revascularization using erectile function recovery as an indicator. As cavernous dysfunction is among the significant causes of ED, erectile function recovery could be judged as almost the mean cavernous function recovery [14]. We determined erectile function recovery using the validated IIEF-6 questionnaire. The control group comprised patients with arteriogenic ED and optimal cavernous function who underwent the same surgery as a certain latency period is present before IIEF-6 normalizes after penile revascularization even in patients with arteriogenic ED and optimal cavernous function.

Our results indicated that penile revascularization could cure ED even when accompanied by cavernous dysfunction. We observed no statistical difference in the degree of cure compared with that in patients with normal cavernous function. In particular, within 1 year after penile revascularization, the degree of erectile function recovery was almost the same in these two groups, suggesting that cavernous function recovered without delay after penile revascularization.

The cumulative efficacy rate of penile revascularization in patients with severe cavernosal dysfunction, i.e. those with a high maintenance flow rate on DICC examination, was relatively low at approximately 50%. However, the other 50% of patients had their ED cured without delay compared with the other patient groups. In other words, even at a severe cavernosal dysfunction degree, approximately half of the patients might be able to recover cavernous function to the same degree as others.

Our surgical results confirmed higher efficacy rates than those of previously reported surgical treatments for mixed vascular ED [17–19], which might be potentially due to the cumulative effective rate calculation using the Kaplan – Meier method, microscope equipment, microsurgical instruments, intraoperative indocyanine green video angiography, and intraoperative blood flow analysis. However, the surgical method we chose might represent the most important difference. We chose the surgical approach of anastomosing the distal end of the inferior epigastric artery end-to-end to the dorsal artery of the penis in all cases. This method is a modification of the penile revascularization procedure reported by Goldstein [8], aiming to restore physiological blood flow to the corpus cavernosum tissue. We think that the recovery mechanism is not only an increase in the amount of blood flow into the cavernous sinus after penile revascularization but also a nutritional supply improvement in the cavernous tissue. According to the anatomical literature [9,10], apart from several helicine arteries that open directly into the sinusoids, some helicine arteries terminate in capillaries and supply blood to the cavernous tissue. In other words, this artery is a feeding vessel for the corpus cavernosum tissue. To supply blood to this circulatory system, improving blood flow in the cavernous arteries is essential. However, only a few studies focused on the revascularization of the dorsal artery of the penis for arteriogenic ED with cavernous dysfunction [17]. Instead, numerous surgeries and treatments have been performed to limit venous outflow, including anastomoses of the hypogastric artery and deep dorsal penile vein, venous ligation, and venous embolization [18,19]. However, all these attempts proved ineffective. We think the ischemic cavernous dysfunction itself should have been treated with penile revascularization rather than addressing the venous leakage that is a result of the cavernous dysfunction.

The results of our study suggest that after penile revascularization surgery, cavernous dysfunction rapidly recovers and optimal function gets restored. This phenomenon is similar to the ‘hibernating myocardium’ in ischemic myocardium, a concept defined as the tissue that survives under chronic ischemia with reduced function and could recover its function as soon as the ischemia improves. Certainly, the cavernous tissue and cardiac muscle differ in various aspects, but this phenomenon is similar to ‘hibernating myocardium’: both are adaptations to a chronic ischemic environment due to reduced blood supply from the feeding arteries. In other words, in an ischemic environment, these organs have reduced function and minimal cell death. Furthermore, revascularization could partially or completely reverse both conditions. We might thus refer to this phenomenon as ‘hibernating cavernosum.’

Cavernous dysfunction may or may not be reversible with penile revascularization. It might be worth investigating whether minimally invasive diagnostic methods used for ‘hibernating myocardium’ diagnosis, such as MRI and PET scans [20], could also be applied to the corpus cavernosum. If diagnosing the potential to cure such conditions would be a possibility before surgery, erectile function could be restored in more patients.

## Adverse events

No serious adverse events occurred during the course of our study. We encountered no cases of glans hyperemia or shortening of the penis. One patient experienced a grade-3 adverse event, i.e. the rupture of the anastomosis due to a trauma that occurred 5 weeks after the surgery, resulting in emergency reoperation. Rigorous guidance regarding anastomosis safety would thus be required.

## Limitation

For safety reasons, we limited the maximum perfusion rate of the DICC test to 120 mL/min, thereby potentially influencing the diagnostic accuracy of the test. Intracavernous injection testing as an objective postoperative test could only be performed in certain cases as patients who regain erectile function after surgery often refuse intracavernous injection testing.

## Conclusion

Penile revascularization using physiological routes in patients with arteriogenic ED and cavernosal dysfunction restored erectile function in certain patients (73.8%). Therefore, cavernous dysfunction appears to be reversible. Erectile function recovery within 12 months after surgery was comparable to that in patients with pure arteriogenic ED, suggesting rapid cavernous function recovery after penile revascularization. This phenomenon appears to be similar to the concept of hibernating myocardium in ischemic myocardium, which we propose to refer to as hibernating cavernosum. The results of this study would potentially expand the indications for penile revascularization.

## Abbreviations

ED: Erectile Dysfunction
IIEF: International Index of Erectile Function
DICC: Dynamic Infusion Cavernosometry and Cavernosography
MRI: Magnetic Resonance Imaging
CTCAE: Common Terminology Criteria for Adverse Events
PET: Positron Emission Tomography
